# Supplementary material for: Cellquant: a vibecoder’s guide to image analysis
Source: bioRxiv. 2026 Sep 3:2026.03.09.710634. Preprint. [Version 2] doi: 10.64898/2026.03.09.710634 (PMC13684596; doi:10.64898/2026.03.09.710634)
Supplement: Supplementary file 1 [file NIHPP2026.03.09.710634v2-supplement-1.pdf]

bioRxiv preprint doi: https://doi.org/10.64898/2026.03.09.710634; this version posted September 3, 2026. The copyright holder for this preprint (which was not certified by peer review) is the author/funder, who has granted bioRxiv a license to display the preprint in perpetuity. It is made available under a CC-BY 4.0 International license.

Supplemental Materials

bioRxiv preprint doi: https://doi.org/10.64898/2026.03.09.710634; this version posted September 3, 2026. The copyright holder for this preprint (which was not certified by peer review) is the author/funder, who has granted bioRxiv a license to display the preprint in perpetuity. It is made available under a CC-BY 4.0 International license.

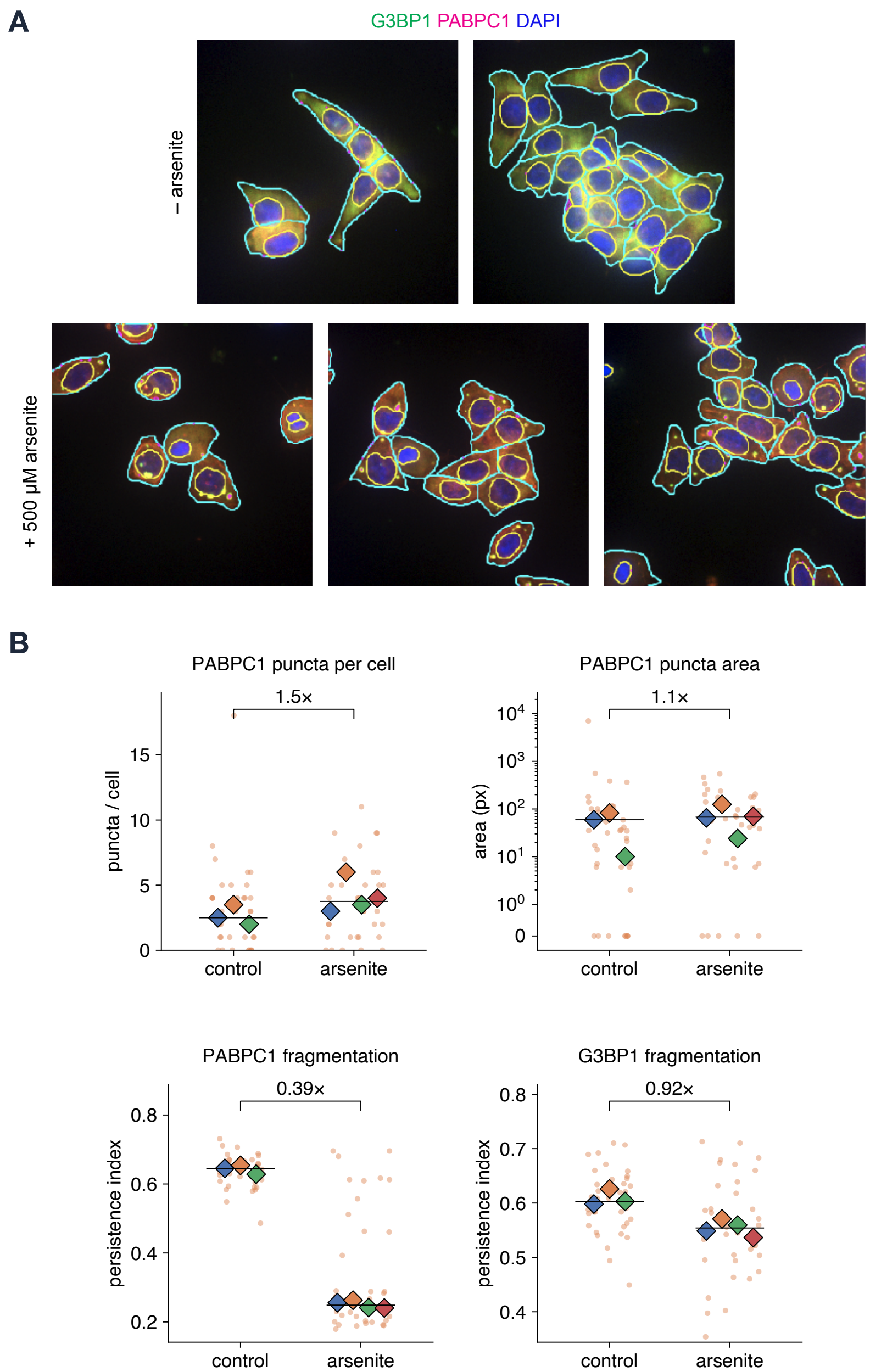

Figure S1: Additional metrics from HCT116 stress granule quantification. (A) Maximum-intensity projections with `cellquant` segmentation overlays: G3BP1 (green), PABPC1 (magenta), and DAPI (blue), untreated (top) or treated with 500 µM sodium arsenite for 1 h (bottom). Cell boundaries are drawn in cyan, nuclear boundaries in yellow, and detected puncta in magenta. **(B)** Per-cell metrics: PABPC1 puncta per cell and puncta area, and the fragmentation index for both channels. Plotting conventions and statistics are as in Fig. 2B, and brackets give the fold change (arsenite / control). The puncta-area panel uses a symmetric-log y axis so that cells containing no puncta remain on the plot at zero. The *fragmentation persistence index* is a threshold-free measure on $[0, 1]$: the signal is swept across all intensity thresholds and the index integrates how persistently it remains broken into separate objects, so it does not depend on choosing any single threshold. Remaining metrics, including the condensate indices, are given in Table S1.

bioRxiv preprint doi: https://doi.org/10.64898/2026.03.09.710634; this version posted September 3, 2026. The copyright holder for this preprint (which was not certified by peer review) is the author/funder, who has granted bioRxiv a license to display the preprint in perpetuity. It is made available under a CC-BY 4.0 International license.

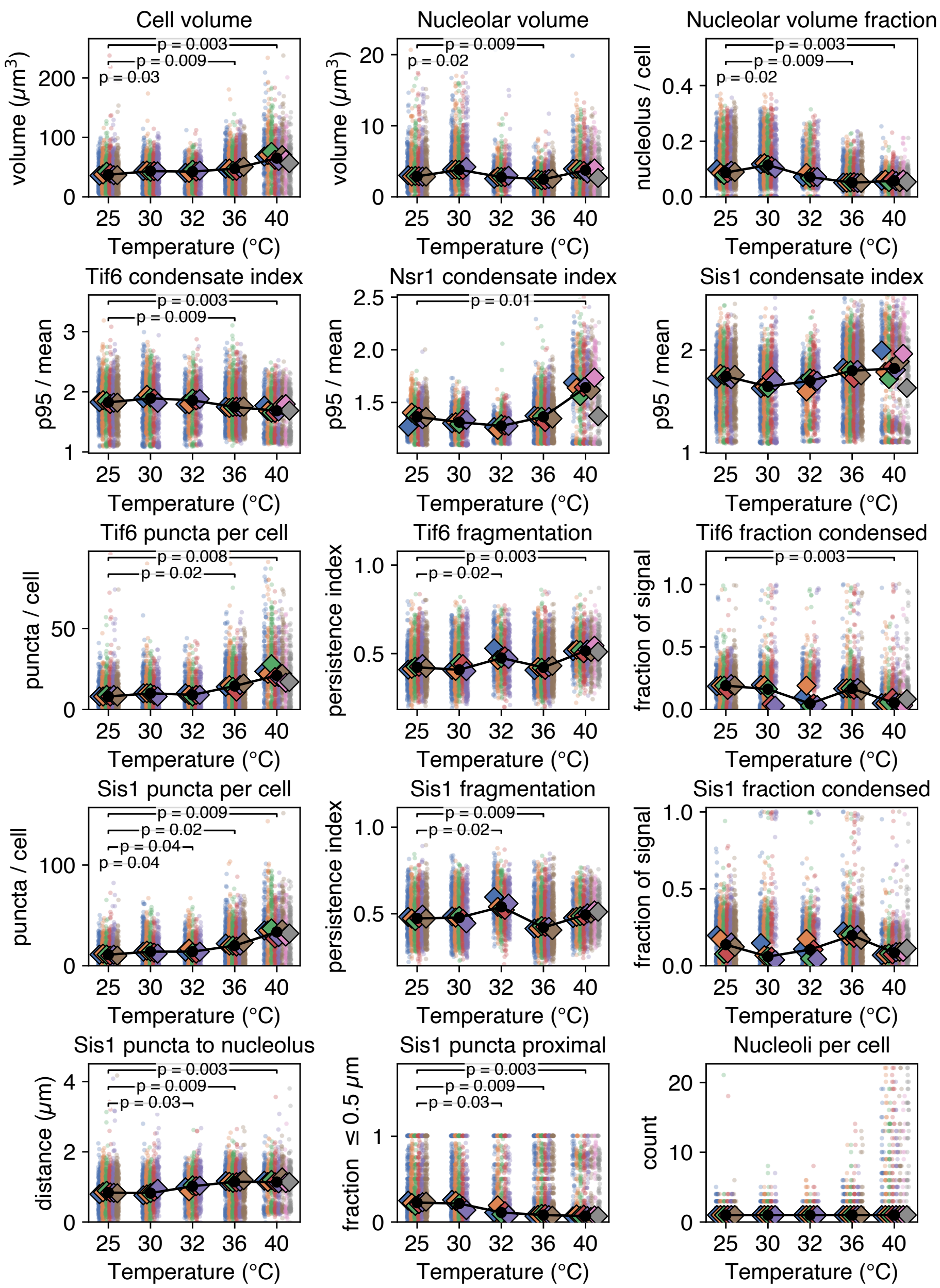

Figure S2: Additional `cellquant` output for the yeast temperature series. Per-cell metrics `cellquant` reports for the yeast series that are not shown in Fig. 3B, computed on the z-stacks. All panels use the conventions of Fig. 3B: individual cells as small points colored by replicate, replicate medians as large diamonds, and the black line joining the median of replicate medians across 8,309 cells from 30 biological replicates. Brackets show Bonferroni-corrected *P*-values against 25°C, and only significant comparisons are drawn. Rows, top to bottom: compartment size (cell volume, nucleolar volume, and nucleolar volume as a fraction of cell volume); condensate index for all three markers (the ratio of the 95th-percentile to the mean intensity within each cell, so values > 1 indicate non-uniform signal); Tif6 puncta count, fragmentation, and fraction condensed; the same three for Sis1; and nucleolar proximity (mean Sis1 puncta-to-nucleolus distance and the fraction of Sis1 puncta within 0.5 μm of the nucleolus) with nucleoli per cell. Nucleolar area, circularity, eccentricity, and solidity are properties of a two-dimensional projection and have no three-dimensional counterpart; nucleolar volume and volume fraction are reported in their place. Manders' M1 and M2 for all three channel pairs are omitted because Fig. 3B already reports colocalization for every pair; they are included in the deposited per-cell tables.

bioRxiv preprint doi: https://doi.org/10.64898/2026.03.09.710634; this version posted September 3, 2026. The copyright holder for this preprint (which was not certified by peer review) is the author/funder, who has granted bioRxiv a license to display the preprint in perpetuity. It is made available under a CC-BY 4.0 International license.

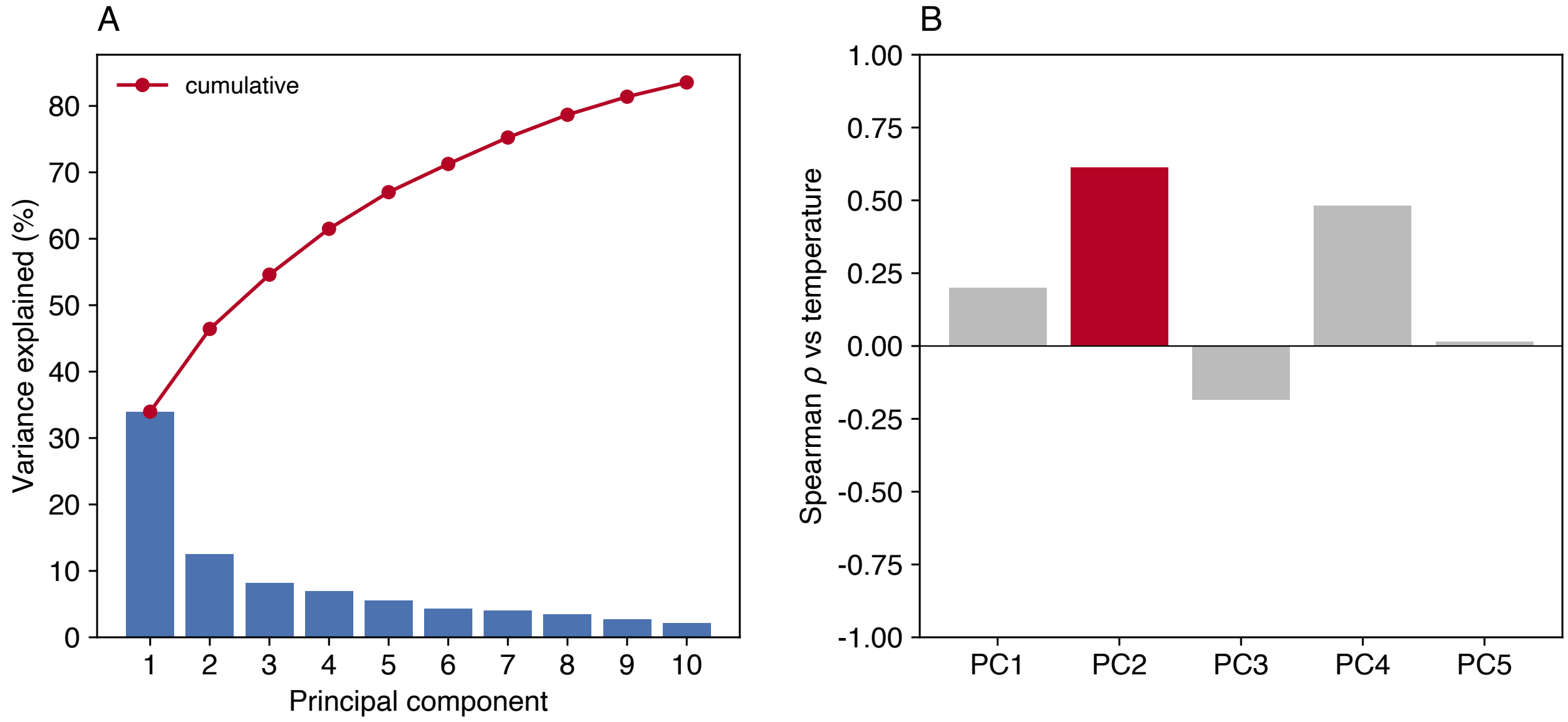

Figure S3: Principal component analysis diagnostics. (A) Variance explained by each of the first ten principal components (bars) and the cumulative total (line). PC1 accounts for 34.0% and the first five components for 67.0% of the variance in the 45-feature matrix. **(B)** Spearman correlation between each of the first five components and growth temperature. Temperature is carried by PC2 ($\rho = +0.61$, highlighted), not by PC1 ($\rho = +0.20$), which is why Fig. 4B plots PC2 against temperature.

bioRxiv preprint doi: https://doi.org/10.64898/2026.03.09.710634; this version posted September 3, 2026. The copyright holder for this preprint (which was not certified by peer review) is the author/funder, who has granted bioRxiv a license to display the preprint in perpetuity. It is made available under a CC-BY 4.0 International license.

Table S1: HCT116 stress granule summary statistics. Values are the median of per-replicate medians. Fold change is arsenite / control; Cliff's δ is the rank effect size belonging to the test performed, bounded $[-1, 1]$, where $+1$ is complete separation. P-values are from two-sided Mann-Whitney tests on replicate medians (control: $n = 3$; arsenite: $n = 4$ replicates). At these sample sizes the smallest attainable two-sided p is 0.057, so this experiment can establish effect sizes but cannot establish significance; the effect-size columns, not the P column, carry the result.

Metric	Control	Arsenite	Fold change	Cliff's δ	P-value
G3BP1 puncta per cell	3	3.5	1.2×	+0.50	0.35
G3BP1 puncta area	27	45.25	1.7×	+0.50	0.40
G3BP1 fraction condensed	0.001537	0.006206	4.0×	+0.67	0.23
PABPC1 fraction condensed	0.001875	0.007728	4.1×	+0.67	0.23
PABPC1 puncta per cell	2.5	3.75	1.5×	+0.75	0.15
G3BP1 condensate index	1.421	1.466	1.0×	+0.50	0.40
PABPC1 condensate index	1.51	1.463	0.97×	−0.33	0.63

bioRxiv preprint doi: https://doi.org/10.64898/2026.03.09.710634; this version posted September 3, 2026. The copyright holder for this preprint (which was not certified by peer review) is the author/funder, who has granted bioRxiv a license to display the preprint in perpetuity. It is made available under a CC-BY 4.0 International license.

Table S2: Yeast temperature series summary. All metrics computed natively on the z-stacks. Values are the median of per-replicate medians across 5–8 biological replicates per temperature. Nucleolar shape descriptors are properties of a projection and have no three-dimensional counterpart; nucleolar volume and equivalent diameter are reported in their place. Statistical comparisons are given in Table S3.

Metric	25°C	30°C	32°C	36°C	40°C
n cells	2088	1527	916	2577	1201
n replicates	6	5	5	6	8
Condensate metrics					
Sis1 condensate index	1.74	1.64	1.70	1.80	1.82
Sis1 frac. condensed	0.137	0.060	0.105	0.197	0.084
Sis1 puncta/cell	11.00	14.00	14.00	20.00	33.50
Tif6 condensate index	1.82	1.89	1.86	1.75	1.68
Tif6 frac. condensed	0.191	0.162	0.046	0.167	0.053
Tif6 puncta/cell	8.00	10.00	9.00	14.50	21.00
Nsr1 condensate index	1.36	1.31	1.28	1.36	1.64
Colocalization					
Pearson R (Tif6 vs Sis1)	0.74	0.77	0.80	0.77	0.66
Pearson R (Tif6 vs Nsr1)	0.82	0.83	0.80	0.72	0.56
Pearson R (Sis1 vs Nsr1)	0.77	0.76	0.75	0.77	0.74
Manders M1 (Tif6 vs Sis1)	0.69	1.00	1.00	1.00	1.00
Manders M1 (Tif6 vs Nsr1)	0.87	0.83	0.83	0.74	0.59
Manders M1 (Sis1 vs Nsr1)	0.84	0.82	0.81	0.76	0.68
Nucleolar proximity					
Sis1 fraction proximal	0.23	0.22	0.11	0.08	0.07
Sis1 mean distance (µm)	0.84	0.82	1.02	1.15	1.14
Tif6 fraction proximal	0.27	0.22	0.12	0.14	0.12
Tif6 mean distance (µm)	0.81	0.90	1.01	1.10	1.07
Cell and nucleolar size					
Cell volume (µm^3)	37.2	43.4	42.5	47.5	65.1
Nucleolar volume (µm^3)	2.88	3.82	2.78	2.48	3.75
Nucleolar eq. diameter (µm)	1.79	2.00	1.76	1.68	1.94

bioRxiv preprint doi: https://doi.org/10.64898/2026.03.09.710634; this version posted September 3, 2026. The copyright holder for this preprint (which was not certified by peer review) is the author/funder, who has granted bioRxiv a license to display the preprint in perpetuity. It is made available under a CC-BY 4.0 International license.

Table S3: Statistical comparisons for the yeast temperature series. Bonferroni-corrected p-values from two-sided Mann-Whitney tests on replicate medians, comparing each temperature to the 25°C reference across the four temperature comparisons. Bold values indicate $p_{adj} < 0.05$. The significant 32°C Tif6–Sis1 Pearson comparison is an *increase* over 25°C; every other significant colocalization change is a decrease.

Metric	30°C	32°C	36°C	40°C
Condensate metrics				
Sis1 condensate index	0.12	0.07	0.26	0.43
Sis1 frac. condensed	0.12	1.00	0.10	0.24
Sis1 puncta/cell	**0.04**	**0.04**	**0.02**	**0.009**
Tif6 condensate index	0.33	1.00	**0.009**	**0.003**
Tif6 frac. condensed	1.00	0.07	0.16	**0.003**
Tif6 puncta/cell	0.25	0.52	**0.02**	**0.008**
Nsr1 condensate index	0.50	0.12	1.00	**0.01**
Colocalization				
Pearson R (Tif6 vs Sis1)	0.07	**0.02**	0.72	0.17
Pearson R (Tif6 vs Nsr1)	0.07	1.00	**0.009**	**0.003**
Pearson R (Sis1 vs Nsr1)	1.00	1.00	1.00	0.43
Manders M1 (Tif6 vs Sis1)	0.90	0.90	1.00	0.97
Manders M1 (Tif6 vs Nsr1)	0.33	0.50	**0.009**	**0.003**
Manders M1 (Sis1 vs Nsr1)	1.00	0.71	**0.03**	**0.003**
Nucleolar proximity				
Sis1 fraction proximal	1.00	**0.03**	**0.009**	**0.003**
Sis1 mean distance (μm)	1.00	**0.03**	**0.009**	**0.003**
Tif6 fraction proximal	1.00	0.09	**0.03**	**0.009**
Tif6 mean distance (μm)	1.00	0.07	**0.009**	**0.003**
Cell and nucleolar size				
Cell volume (μm^3)	**0.03**	0.07	**0.009**	**0.003**
Nucleolar volume (μm^3)	**0.02**	0.33	**0.009**	0.08
Nucleolar eq. diameter (μm)	**0.02**	0.33	**0.009**	0.08

bioRxiv preprint doi: https://doi.org/10.64898/2026.03.09.710634; this version posted September 3, 2026. The copyright holder for this preprint (which was not certified by peer review) is the author/funder, who has granted bioRxiv a license to display the preprint in perpetuity. It is made available under a CC-BY 4.0 International license.

Table S4: `cellquant` **parameters.** Complete parameter settings used for the HCT116 mammalian and yeast temperature series analyses. Parameters not listed used default values. The HCT116 analysis is two-dimensional and its size parameters are therefore in pixels; the yeast analysis is three-dimensional and its size parameters are in voxels, which are the quantities the three-dimensional code path uses. Because a Z crop was applied to the yeast stacks, cells whose masks touch the top or bottom plane of the cropped volume were excluded so that axially truncated cells did not enter the per-cell measurements.

Parameter	HCT116 analysis	Yeast analysis
Analysis mode	2D (MIP)	3D (z-stack)
Cell type preset	mammalian	yeast
Cellpose model	cpsam	cpsam
3D seg. method	n/a	slice stitching, IoU 0.65
Voxel size (µm)	n/a (pixel units)	0.10571 lateral, 0.23 axial
Z crop (planes)	n/a	41, axially centred
Seg. downsample	3×	2×
Cell diameter (px)	auto	40
Seg. channel	PABPC1	composite
Cell size filter	default	1,500–200,000 vox
Cellprob threshold	0.0	−1.0
Puncta channels	G3BP1, PABPC1	Sis1, Tif6
Puncta compartment	cytosol	whole-cell
LoG sigma	2.0	1.5
Puncta min size	6 px	4 vox
Puncta max size	10,000 px	3,000 vox
Threshold method	Otsu	Otsu
Nuclear req.	1–4 nuclei	none
Nucleus dilation (px)	3	0
Colocalization	no	yes (whole-cell, native 3D)
Nucleolar proximity	no	yes (Nsr1, 0.5 µm)
Plot style	superplot	trend
